# Prenatal and Postnatal Diagnosis and Genetic Background of Corpus Callosum Malformations and Neonatal Follow-Up

**DOI:** 10.3390/children11070797

**Published:** 2024-06-28

**Authors:** Virág Bartek, István Szabó, Ágnes Harmath, Gábor Rudas, Tidhar Steiner, Attila Fintha, Nándor Ács, Artúr Beke

**Affiliations:** 1 Department of Obstetrics and Gynecology, Semmelweis University, 1085 Budapest, Hungary; bartek.virag@semmelweis.hu (V.B.); szabo.istvan@semmelweis.hu (I.S.); harmath.agnes@semmelweis.hu (Á.H.); steiner.tidhar@semmelweis.hu (T.S.); acs.nandor@semmelweis.hu (N.Á.); 2 Heim Pál National Pediatric Institute, 1089 Budapest, Hungary; rudas.gabor@semmelweis.hu; 3 Department of Pathology and Experimental Cancer Research, Semmelweis University, 1085 Budapest, Hungary; fintha.attila@semmelweis.hu

**Keywords:** corpus callosum, ventriculomegaly, developmental neurology, ultrasound

## Abstract

Introduction: The corpus callosum is one of the five main cerebral commissures. It is key to combining sensory and motor functions. Its structure can be pathological (dysgenesis) or completely absent (agenesis). The corpus callosum dys- or agenesis is a rare disease (1:4000 live births), but it can have serious mental effects. Methods: In our study, we processed the data of 64 pregnant women. They attended a prenatal diagnostic center and genetic counseling from 2005 to 2019 at the Department of Obstetrics and Gynecology at Semmelweis University. Results: The pregnancies had the following outcomes: 52 ended in delivery, 1 in spontaneous abortion, and 11 in termination of pregnancy (TOP) cases (n = 64). The average time of detection with imaging tests was 25.24 gestational weeks. In 16 cases, prenatal magnetic resonance imaging (MRI) was performed. If the abnormality was detected before the 20th week, a genetic test was performed on an amniotic fluid sample obtained from a genetic amniocentesis. Karyotyping and cytogenetic tests were performed in 15 of the investigated cases. Karyotyping gave normal results in three cases (46,XX or XY). In one of these cases, postnatally chromosomal microarray (CMA) was later performed, which confirmed Aicardi syndrome (3q21.3–21.1 microdeletion). In one case, postnatally, the test found Wiedemann–Rautenstrauch syndrome. In other cases, it found X ring, Di George syndrome, 46,XY,del(13q)(q13q22) and 46,XX,del(5p)(p13) (Cri-du-chat syndrome). Edwards syndrome was diagnosed in six cases, and Patau syndrome in one case. Conclusions: We found that corpus callosum abnormalities are often linked to chromosomal problems. We recommend that a cytogenetic test be performed in all cases to rule out inherited diseases. Also, the long-term outcome does not just depend on the disease’s severity and the associated other conditions, and hence proper follow-up and early development are also key. For this reason, close teamwork between neonatology, developmental neurology, and pediatric surgery is vital.

## 1. Introduction

The incidence of corpus callosum agenesis, i.e. the absence of the corpus callosum, is 1:4000 live births. In terms of its etiology, 30–45% of cases are due to genetic causes, 10% are due to chromosomal disorders, and 20–35% are associated with a syndrome. No literature data is available on what percentage of cases of intrauterine infection is responsible for the development of corpus callosum dys/agenesis. In some cases, environmental factors (such as maternal alcohol consumption) also lead to corpus callosum agenesis [1].

The corpus callosum is one of the five major cerebral commissures. It is one of the largest tracts of white matter in the brain. Its role is to connect the right and left cerebral hemispheres. According to assumptions, 2–3% of brain fibers pass through it. Its main job is to coordinate the brain’s hemispheres. It also integrates sensory and motor functions [2]. The corpus callosum is suggested to be a major epileptogenic pathway due to its role [3].

Isolated corpus callosum agenesis is a disorder compatible with life. However, about 25% of fetuses with antenatally diagnosed isolated corpus callosum agenesis have later mental problems. However, even with normal intelligence, a mild deficit in learning or social skills may occur [2]. If the disease is part of a syndrome, the outcome depends on the specific syndrome.

Known chromosomal mutations affect chromosomes 1, 4, 6, 8 and 17. Deletion is the most common mutation. The 1q42–q44 deletion causes varying severity of corpus callosum agenesis [2]. Most of the proteins coded by the affected regions regulate or take part in a key moment of nervous system development, and so often, other parts of the nervous system are affected as well, presented as microcephaly, hydrocephalus, or craniofacial abnormalities.

In terms of inheritance, autosomal dominant, recessive and X-chromosome-linked syndromes are also known.

In part of the cases, Aicardi syndrome is a very severe form of X-chromosome-linked dominant (XLD) inheritance, which is incompatible with life in male fetuses, but early mortality is also high in female fetuses. In addition to corpus callosum agenesis, it is associated with infantile seizures (infantile spasm) and the development of chorioretinal lacunae [4]. In other cases, the Aicardi syndrome is associated with 3q21.3–21.1 microdeletion.

An autosomal dominant form is frontonasal dysplasia, Goldenhar syndrome, Kallmann syndrome or neurofibromatosis; the autosomal recessive form is Andermann syndrome, craniotelencephalic dysplasia, Da Silva or Leigh syndrome. Isolated corpus callosum agenesis can be inherited as autosomal recessive, X-linked recessive, or autosomal recessive [5].

The main mutations leading to corpus callosum abnormalities are described in detail in Table 1. Unless otherwise indicated, the source of all data shown there is from Online Mendelian Inheritance in Man, OMIM^®^. McKusick-Nathans Institute of Genetic Medicine, Johns Hopkins University Online Gene Database [6].

In recent years, magnetic resonance imaging (MRI) has gained more and more space in prenatal diagnostics. Prenatal MRI can confirm and clarify the prenatal ultrasound diagnosis. The fetal corpus callosum can best be examined in the axial plane. Indirect signs are the absence of the cavum septi pellucidi, the enlargement or cystic change of the arachnoid plexus, the drop-like change of the posterior horn of the lateral cerebral ventricle, and the widening of the lateral cerebral ventricle [7]. In the study by Ibrahim et al., where prenatal MRI was examined in the diagnosis of corpus callosum agenesis, they found that it was 75% more effective than a traditional 2D ultrasound. In their study, the sensitivity was 100% and specificity was 67%, with a positive predictive value of 96%. It should be noted that their results were based on 27 examined cases, so it cannot be considered a study with a large number of cases; however, since corpus callosum abnormalities are rare anyway, it is difficult to carry out a larger, comprehensive study with a larger number of cases in order to establish sensitivity [8]. In conclusion, MRI examination can confirm or refute the ultrasound findings with great certainty [9]. 

Malformation of the corpus callosum is difficult to detect before the 18th gestational week [2]. On ultrasound, colpocephaly, high-lying enlarged third ventricle and Texan-Longhorn configuration can be observed in addition to the absence of the corpus callosum [1]. These can confirm the suspicion, as the ultrasound always leaves doubt, that the absence of a formula might just be due to the fetus’s position or the technician’s fault. In their 2012 study, Santo et al [9] found that the rate of false positive ultrasound findings is 20%. 

**Table 1:** Corpus Callosum associated with syndromes.

Name of the Syndrome	Type	Associated Defects	Coding Gene	Full Name of the Gene	Inheritance	Locus
Acrocallosal syndrome [10,11]	Type Shinzel	anencephaly, agenesis of corpus callosum, mental retardation, polydactyly, ventriculomegaly, and Dandy–Walker malformation	KIF7	kinesin family member 7	AR	15q26.1
			GLI3	GLI family zinc finger 3	AR	7p14.1
Apert syndrome [12]	Type I	encephalocele, mental retardation, craniosynostosis, agenesis of corpus callosum, hypertelorism, maxilla hypoplasia, and ventriculomegaly	FGFR2	fibroblast growth factor receptor 2	AD	10q26.13
Aicardi syndrome [4]		IUGR, Dandy–Walker malformation, cataract, early puberty, hiatus hernia, spina bifida, vermis hypoplasia, polymicrogyria, choroid plexus cyst, cavum septum pellucidum, Chiari-malformation, microcephaly, and teratoma	3q21.3–21.1 deletion or X/3 unbalanced transloc		AD XD	3q21.3–21.1
Cerebrofaciothoracic dysplasia [13]		short stature, macrocephaly, brachycephaly, hypertelorism, low-set ears, hypoplasia of corpus callosum, ventriculomegaly, and polyhydramnios	TMCO1	transmembrane and coiled-coil domains 1	AR	1q24.1
Cleft lip/palate with abnormal thumbs and microcephaly [14]		short stature, hypertelorism, horseshoe kidney, microcephaly, abnormalities of the fingers, and agenesis of corpus callosum (?)	ESCO2	establishment of sister chromatid cohesion N-acetyltransferase 2	AR	8p21.1
Coffin–Siris syndrome [15,16,17,18,19,20,21,22]		IUGR, mental retardation, Dandy–Walker malformation, structural heart abnormalities, scoliosis, and clinodactyly	SOX11	SRY-box transcription factor 11	AD	2p25.2
			SMARCD1	SWI/SNF related, matrix associated, actin dependent regulator of chromatin, subfamily d, member 1	AD	12q13.12
			ARID1B	AT-rich interaction domain 1B	AD	6q25.3
			SMARCA4	SWI/SNF related, matrix associated, actin dependent regulator of chromatin, subfamily a, member 4	AD	19p13.2
			SMARCC2	SWI/SNF related, matrix associated, actin dependent regulator of chromatin subfamily c member 2	AD	12q13.2
			SOX4	SRY-box transcription factor 4	AD	6p22.3
			ARID2	AT-rich interaction domain 2	AD	12q12
			DPF2	double PHD fingers 2	AD	11q13.1
			SMARCE1	SWI/SNF related, matrix associated, actin dependent regulator of chromatin, subfamily e, member 1	AD	17q21.2
			SMARCB1	SWI/SNF related, matrix associated, actin dependent regulator of chromatin, subfamily b, member 1	AD	22q11.23
			ARID1A	AT-rich interaction domain 1A	AD	1p36.11
Christianson syndrome [23]		long, narrow face, macrotia, strabism, pectus excavatum, adducted thumb, thick eyebrows, cerebellar atrophy, a/dysgenesis of corpus callosum, ventriculomegaliy, and microcephaly	SLC9A6	solute carrier family 9 member A6	XR	Xq26.3
Crane–Heise syndrome [24]		IUGR, micrognathia, hypertelorism, low-set ears, penis hypoplasia, syndactyly, ventriculomegaly, and agenesis/hypoplasia of corpus callosum	not known	not known	AR	not known
Craniotelencephalia syndrome [25]		encephalocele, craniosynostosis, agenesis of corpus callosum, mental retardation, and micocephalia	not known	not known	AR	not known
Dincsoy–Salih-Patel syndrome [26]		short stature, hypopituarism, abnormalities of the eye, hypoplasia/agenesis of septum pellucidum, a/dysgenesis of corpus callosum, and structural heart abnormalities	HESX1	HESX homeobox 1	AD/AR	3p14.3
			SOX2?	SRY-box transcription factor 2		3q26.33
			SOX3?	SRY-box transcription factor 3		Xq27.1
			OTX2?	orthodenticle homeobox 2		14q22.3
Focal dermal aplasia/hypoplasia [27]		short stature, low-set ears, teleangiectasia, horseshoe kidney, hydronephrosis, abnormalities of the genital tract, oligodactyly, omphalocele, agenesis of corpus callosum, spina bifida, Chiari malformation, microcephaly, hydrocephalus	PORCN	porcupine O-acyltransferase	XD	Xp11.23
Frontonasalis dysplasia [28]	acromelic type	encephalocele, hypertelorism, cranium bifidum occultum anterior, agenesis of cerebellar vermis, Dandy–Walker malformation, and agenesis of corpus callosum	ZSWIM6	zinc finger SWIM-type containing 6	AD	5q12.1
Goldenhar complex [29]		encephalocele, abnormalities of the face, the ear and the spine, mental retardation, Chiari malformation, hydrocephalus, and renal agenesis	not known	not known	AD?	not known
Gorlin–Goltz syndrome (Nevoid basal cell carcinoma syndrome) [30]		spina bifida, basal-cell carcinoma, macrocephaly, hypertelorism, and other abnormalities of the nervous system	PTCH1	patched 1	AD	9q22.32
Hydrolethalus syndrome [31]		encephalocele, hydrocephalus, osetochondroplasia, cleft palate/lips, abnormalities of the limbs, polyhydramnios, anencephaly, and ventriculomegaly	HYLS1	HYLS1 centriolar and ciliogenesis associated	AR	11q24.2
Kapur–Toriello syndrome [32]		low-set ears, tetralogy of Fallot, ventricular septal defects, intestinal malrotation, penis hypoplasia, polymicrogyria, and dysgenesis of corpus callosum	not known	not known	AR	not known
Leigh syndrome [33]		hepatocellular necrosis, abnormalities of the eye, and agenesis of corpus callosum	not known	not known	AR	not known
Leprechaunism [34]		IUGR, short stature, low-set ears, hypertelorism, cardiomyopathy, hepatomegaly, enlarged kidney, genital abnormalities, large limbs, microcephaly, and agenesis of corpus callosum	INSR	insulin receptor	AR	19p13.2
Lujan–Fryns syndrome [35]		tall stature, macrocephaly, micrognathia, long nose, maxilla hypoplasia, low-set ears, septal defects, pectus excavatum, arachnodactyly, and agenesis of corpus callosum	MED12	mediator complex subunit 12	XR	Xq13.1
Marshall–Smith syndrome [36]		tall stature, low-set ears, atrial septal defect, pectus excavatum, omphalocele, macrogyria, agenesis of corpus callosum, and cerebral atrophy	NFIX	nuclear factor I X	AD	19p13.13
Meckel syndrome [37,38,39,40,41,42,43,44,45,46,47]		anencephaly, encephalocele, vermis hypoplasia, multicystic kidney dysplasia, polydactyly, oligohydramnios, pulmonary hypoplasia, and Dandy–Walker malformation	B9D1	B9 domain containing 1	AR	17p11.2
			B9D2	B9 domain containing 2	AR	19q13.2
			CC2D2A	coiled-coil and C2 domain containing 2A	AR	4p15.32
			CEP290	centrosomal protein 290	AR	12q21.32
			MKS1	MKS transition zone complex subunit 1	AR	17q22
			RPGRIP1L	RPGRIP1 like	AR	16q12.2
			TCTN2	tectonic family member 2	AR	12q24.31
			TMEM67	transmembrane protein 67	AR	8q22.1
			TMEM107	transmembrane protein 107	AR	17p13.1
			TMEM216	transmembrane protein 216	AR	11q13.1
			TMEM231	transmembrane protein 231	AR	16q23.1
Neu–Laxova syndrome [48]		IUGR, Dandy–Walker malformation, abnormalities of the mouth, macrotia, hypertelorism, proptosis, hypogonadism, large hands, ichthyosis, macrogyria, absent septum pellucidum, lissencephaly, cerebellar hypoplasia, polymicrogyria, microcephaly, and polyhydramnios	PSAT1	phosphoserine aminotransferase 1	AR	9q21.2
			PHGDH	phosphoglycerate dehydrogenase	AR	1p12
Rubinstein–Taybi syndrome [49]		short stature, micrognathia, low-set ears, hypertelorism, brachydactyly, microcephaly, agenesis of corpus callosum, polyhydramnios, and hemangioma	not known	not known	AD?	not known
Smith–Lemli-Opitz syndrome [50]		short stature, micrognathia, dental abnormalities, low-set ears, septal defects, ptosis, pulmonary hypoplasia, penis hypoplasia, hypospadias, polydactyly, dermatoglyphics, photosensitivity, cerebellum hypoplasia, a/dysgenesis of corpus callosum, ventriculomegaly, microcephaly, and polyhydramnios	DHCR7	7-dehydrocholesterol reductase	AR	11q13.4
Pyruvate dehydrogenase deficiency (pyruvate dehydrogenase complex deficiency—PDCD) [51,52,53]		IUGR, lactic acidosis, hyperammonaemia, facialis dysmorphy (narrow head, prominent forehead, wide nasal bridge), neurological impairments (intellectual impairments, ataxia, abnormal eye movements, blindness), cranial abnormailities (microcephaly, corpus callosum dysgenesis, cerebral cortex atrophy, brain laesions), and muscular abnormalities (hypotonia, spasticity, ataxia)	PDHA1	pyruvate dehydrogenase E1 subunit alpha 1	XD	Xp22.12
			PDHB	pyruvate dehydrogenase E1 subunit beta	AR	3p14.3
			PDHX	pyruvate dehydrogenase complex component X	AR	11p13
Warburg syndrome [54,55,56,57,58,59,60,61]		Dandy–Walker malformation, macrocephaly, optic atrophy, muscle weakness, macrogyria, a/dysgenesis of corpus callosum, absent septum pellucidum, lissencephaly, cerebellar hypoplasia, ventriculomegaly, polymicrogyria, abnormality of neuronal migration, hydrocephalus, and encephalocele	B3GALNT2	beta-1,3-N-acetylgalactosaminyltransferase 2	AR	1q42.3
			B4GAT1	beta-1,4-glucuronyltransferase 1	AR	11q13.2
			DAG1	dystroglycan 1	AR	3p21.31
			FKRP	fukutin related protein	AR	19q13.32
			FKTN	fukutin	AR	9q31.2
			CRPPA	CDP-L-ribitol pyrophosphorylase A	AR	7p21.2
			POMT1	protein O-mannosyltransferase 1	AR	9q34.13
			POMT2	protein O-mannosyltransferase 2	AR	14q24.3
			POMGNT1	protein O-linked mannose N-acetylglucosaminyltransferase 1 (beta 1,2-)	AR	1p34.1
			POMK	protein O-mannose kinase	AR	8p11.21
Wolf–Hirschhorn syndrome [62,63]		short stature, microcephaly, micrognathia, short philtrum, prominent glabella, ocular hypertelorism, dysplastic ears and periauricular tags, corpus callosum dysgenesis, intellectual disability, muscle hypotonia, seizures, and congenital heart defects	4p16.3 deletion			4p16.3 deletion
			NSD2	nuclear receptor binding SET domain protein 2	AR	4p16.3
			LETM1	leucine zipper and EF-hand containing transmembrane protein 1	AR	4p16.3

Abbreviation: autosomal recessive (AR), autosomal dominant (AD), X-linked dominant (XD), X-linked recessive (XR) and intrauterine growth retardation (IUGR).

## 2. Materials and Methods

During our retrospective study, we processed data from 64 pregnant women, whose pregnancy was affected by corpus callosum malformations. They attended genetic counseling between 2005 and 2019 at the Baross Street Department of the Semmelweis University Obstetrics and Gynecology Clinic. The sources of the data are genetic records, fetopathological findings, and the MedSol system. A written consent was obtained.

The follow-up is complicated by the fact that the pregnant women participating in genetic counseling could apply for termination of pregnancy or delivery at the regional obstetrics and gynecology clinic, so in such cases, we did not have the results of fetopathology or neonatal examinations. The autopsy findings were provided by the Semmelweis University Institute of Pathology (Department I).

Ultrasound examinations were performed in the Ultrasound Laboratory of the Semmelweis University Obstetrics and Gynecology Clinic, Baross Street Department, Medison Sonoace X8 (Medison Co., Ltd., Seoul, Republic of Korea), Samsung Medison UGEO H60 (Samsung Medison Co., Ltd., Seoul, Republic of Korea), Samsung Medison WS80A (Samsung Medison Co., Ltd.), and Philips^®^ HD 11XE (Philips Ultrasound, Amsterdam, The Netherlands). MRI examinations were performed in coronal T2 TSE, sagittal T2 TSE, and axial T2 TSE plane with Philips Ingenia 3 Tesla equipment.

Karyotyping was performed by amniocentesis (GAC) and chorionic villus sampling (CVS). Amniotic fluid and chorionic villus samples went under cytogenetic processing. In some of the cases, the detection of numerical chromosomal abnormalities was also confirmed by molecular genetic testing, using the quantitative fluorescence-PCR (QF-PCR) technique.

Data recording and simple statistical analysis were performed with Microsoft Excel for Windows (version number: 16.0.13231.20250 64-bit).

## 3. Results

The average age of the 64 examined pregnant women was 30.475 ± 6.56 (no data available in five cases, n = 59), and their median age was 30. Regarding the outcome of the pregnancies, the pregnancy ended in 52 cases with delivery, in 1 case with spontaneous abortion, and in 11 cases with termination of pregnancy (n = 64). Of the pregnancies, 63 were singular, while one was a gemini (“B” fetus). The average time to terminate the pregnancy was 20.45 ± 1.61 completed gestational weeks (n = 11), while the median time was 20 weeks (n = 11). In the case of deliveries, the average birth week was 35.62 ± 3.54, and the median gestational week was 37 (n = 52). The spontaneous abortion took place in the 19th gestational week. There were 33 female and 27 male fetuses in our study (n = 60, no data in 4 cases).

Compared to the percentile table of the World Health Organization, the average weight percentiles adjusted for gestational week and gender are 26.19 percentiles (n = 59, no data available in five cases), and the median is 7.4 percentiles. In 23 cases, the weight was below the 2.5th percentile (intrauterine growth retardation).

All 64 cases underwent at least one prenatal ultrasound examination at our Clinic, and 16 cases underwent a prenatal MRI examination.

The examined cases were divided into three groups. We considered the case fully diagnosed prenatally if the prenatal tests (ultrasound or MRI diagnosis) recognized the corpus callosum dys/agenesis and was later confirmed by the neonatological and autopsy findings. We considered the case partially diagnosed if the prenatal examinations raised the possibility of corpus callosum dys/agenesis, but the postnatal examinations also found other malformations. Cases where corpus callosum dys/agenesis were not diagnosed by prenatal examination were considered undiagnosed.

In cases detected by ultrasound, the average week of detection was 25.24 gestational weeks (n = 64, no data available in three cases).

**Figure 1:**
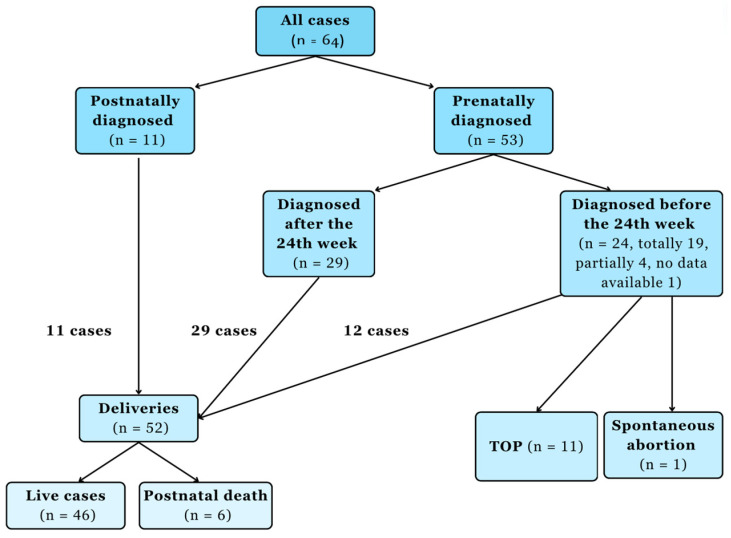
Chart flow of the corpus callosum malformation cases. Abbreviation: termination of pregnancy (TOP).

Of the investigated cases, the corpus callosum deviation was diagnosed in 53 cases by ultrasound (Figure 1). 

In 45 cases there were a full diagnosis of the corpus callosum abnormality prenatally. In eight cases, the ultrasound provided a partial diagnosis, i.e. other abnormalities affecting the nervous system were described, but the corpus callosum abnormality was not described; however, it was suspected. The diagnosis was a subarachnoid cyst in two cases, ventriculomegaly in four cases, and microcephaly in two cases. In 11 cases, corpus callosum agenesis was diagnosed postnatally. In one case, with a postnatal ultrasound scan, in another, with a postnatal MRI scan. In one, an autopsy raised a strong suspicion. In eight, neonatological findings revealed the diagnosis (Table 2).

Due to the difficulty of follow-up, we managed to confirm the prenatal diagnosis in 37 cases with postnatal follow-up. In four cases, the birth happened outside of our hospital.

Karyotyping and cytogenetic tests were performed in 15 of the investigated cases. Karyotyping gave normal results in three cases (46,XX or XY). In one of these cases, CMA was later performed postnatally, which confirmed Aicardi syndrome (3q21.3–21.1 microdeletion). In one case postnatally, Wiedemann-Rautenstrauch syndrome, in one case X ring, in one case Di George syndrome, in one case 46,XY,del(13q)(q13q22), and in one case 46,XX,del(5p)(p13) (Cri-du-chat syndrome) was diagnosed. Edwards syndrome was found in six cases and Patau syndrome in one case.

In 46 cases, there was a live birth, or the newborn reached the age of one year. Long-term (3 years) follow-up was performed in 20 cases. One child developed according to his age. In 15 cases, the child was mentally affected (moderately severe retardation and slow development). In 5 cases, the locomotor system was affected. Four children became epileptic, requiring treatment (Table 3).

The table was based on the Online Mendelian Inheritance in Man, OMIM^®^. McKusick-Nathans Institute of Genetic Medicine, Johns Hopkins University (Baltimore, MD, USA) [6]. The specific gene mutations are cited above.

## 4. Discussion

Agenesis or dysgenesis of the corpus callosum is rare; however, due to the frequent genetic involvement and even severe mental involvement, diagnosing it accurately and quickly is crucial.

We assessed the weight development of the fetuses included in our study. We found that in the studied population, corrected for gestational week, the fetuses were on average at the 26.19th percentile, i.e. there was no intrauterine growth retardation/small for gestational age (IUGR/SGA), but their weight development was close to the lower limit of the normal range. In 25 cases, the diagnosis of IUGR/SGA was confirmed. In their 2014 article, Egaña-Ugrinovic et al investigated the relationship between low weight, corpus callosum abnormalities and neurological complications. In total, 117 IUGR and 73 control fetuses were examined with MRI, and then their neurological development was examined based on the Neonatal Behavioral Assessment Scale. In fetuses affected by IUGR, the corpus callosum was significantly smaller (total CC Area IUGR: 1.3996 ± 0.26 vs. AGA: 1.664 ± 0.31; *p* < 0.01), and they also performed worse on neurological tests. Although genetic testing was not carried out in their research, it can be seen that lower weight in itself delays the development of the corpus callosum. Whether this lower weight is a cause, or a consequence requires further research. However, it can be seen that lower birth weight has a negative effect on the development of the corpus callosum. Our study also supports the relationship between corpus callosum abnormalities and low birth weight, but its clinical relevance is still questionable, and it is definitely an interesting area that requires further investigation [64].

Based on the literature data, it is hard to diagnose the disease before the 18th week of pregnancy. During a systematic review, Zhang et al. examined 12 articles in which the prenatal ultrasound diagnosis of corpus callosum abnormalities was examined. A sensitivity of 72% and a specificity of 98% were found for agenesis of the corpus callosum. For obvious reasons, both specificity and sensitivity increased as pregnancy progressed [65]. In our study, we obtained a sensitivity of 82.81% based on the ultrasound diagnosis, which is a little bit higher than in the systematic review carried out by Zhang et al. This may be due to the fact that we worked with a much smaller number of cases than Zhang et al.’s meta-analysis (64 vs. 544 cases), but in terms of proportions, we also obtained a similar result.

Shwe et al. processed 127 corpus callosum dys/agenesis cases in a study similar to ours. The follow-up was performed in 72 cases, of which a postnatal MRI examination was performed in 59 cases. In 73% (43 of 59 of cases), the prenatal diagnosis was confirmed by MRI [66]. In their study, there were 75 live births, of which 62 were followed up. Intelligence was affected in 26 cases (47%), in our investigation in 15 cases (75%), and epilepsy in 13 cases (22%), while in our investigation in 4 cases (20%). In Shwe et al.’s study, complete corpus callosum agenesis had more serious consequences, but we did not investigate this. Shwe et al also found 18 trisomies among their studied cases. In their study, the genetic screening was negative in 57 cases (44.8%), while in our study this rate is higher, 80%. From the above, it can be seen that corpus callosum abnormalities are a very heterogeneous disease, both in terms of genotype and phenotype. This, as well as the different number of cases, may account for the differences between Shwe et al.’s and our research.

However, both studies show that both the genetic ultrasound screening performed at week 20 and the prenatal MRI are very important tools in the diagnosis of abnormalities affecting the corpus callosum. Also, it can be seen that there is a clear correlation between corpus callosum deviations, developmental delay, lower intelligence level and epilepsy.

The 46,XY,del(13q)(q13q22) mutation was described by Turleau et al. in 1983 in connection with a case report [67]. In addition to corpus callosum agenesis, bilateral retinoblastoma and dysmorphic face were present. In the case we found, in addition to corpus callosum agenesis, the dysmorphic face was present, as well as persistent superior vena cava sinistra and oligodactyly. Chorioretinal coloboma was present among ophthalmic abnormalities. Cryptorchidism was diagnosed after birth. There was no long-term follow-up, so we have no information about the retinoblastoma in our case. 

About chromosomal abnormalities, it is difficult to give an exact figure as to what proportion of individual chromosomal abnormalities are associated with corpus callosum dys or agenesis. In a systematic review, Santo et al. found that the number of chromosomal abnormalities was 17.8 percent, but they did not differentiate between isolated and complex corpus callosum agenesis [9]. It is a well-supported fact in the literature that the Edwards and Patau syndromes (we also found) are often associated with corpus callosum abnormalities, but we did not find any relevant literature on the proportion of this. In the review by Cereda et al., corpus callosum abnormalities were present in 5–25% of the Edwards syndrome cases; there is no more precise data [68]. Corpus callosum abnormalities have been described in most cases of Patau syndrome, but there is no precise literature data (it should be noted that since the syndrome is lethal, due to modern screening methods, the affected often terminate the pregnancy before the second genetic ultrasound) [69,70,71].

Based on the literature data, in 25% of the cases, mental functions are subsequently affected at a certain level [72]. In our study, we found a higher rate; however, due to the small number of cases, this cannot be considered a significant result either. Our results are also influenced by the fact that in 11 cases, the pregnancy was terminated, and in 6 cases, the newborn died perinatally, but due to the severe neural and associated disorders, it can be assumed that the mental functions were at least moderately, rather than severely, affected.

In addition, in the past few years, the differences affecting the corpus callosum have come into focus in the background of psychiatric disease research. The relationship between corpus callosum agenesis and psychosis, bipolar and unipolar depression is suggested [73]. The underlying pathomechanism is still the subject of further research [74]. 

However, it is possible that children who are currently living with normal neurological function according to our follow-up may later develop other neurological abnormalities, and their further examination may open up exciting avenues in the future.

## 5. Conclusions

Based on the literature data, due to the location of the corpus callosum, the ultrasound examination can give false results in 25% of the cases; therefore, in case of suspicion, a prenatal MRI examination is recommended. This is particularly important, as a proven corpus callosum agenesis can lead to the termination of the pregnancy based on genetic indications. Therefore, not only a timely but also a very accurate diagnosis is particularly important, as this allows the pregnant woman to make an informed decision. If it is no longer possible to terminate the pregnancy, or if the pregnant woman wishes to continue the pregnancy, accurate MRI diagnostics can allow the allied professions (especially colleagues from developmental neurology, pediatric neurology, neonatology and pediatric surgery) to develop an appropriate therapeutic and long-term follow-up plan, thus giving the child the best possible care.

Due to the genetic etiology, chromosomal analysis of affected families and children is necessary, as some of the pathologies are inherited by dominant, recessive, or sex chromosomes.

It can also be seen from the above that due to the heterogeneous etiology, genetic and environmental reasons, the impact of corpus callosum dys/agenesis on the quality of life and mental functions is difficult to assess prenatally; however, accurate imaging and molecular genetic tests can help the affected pregnant woman in decision making, and later, the colleagues providing postnatal care.

The disease is rare in the first place, so collecting significant data is difficult. However, for the sake of obtaining a larger amount of data for processing, in the future, it is necessary to strive for more precise follow-ups and to conduct prospective studies, if necessary, involving genetics and neonatology co-specialties.

## Figures and Tables

**Table 2:** Effectiveness of ultrasound diagnosis of corpus callosum abnormalities.

Fully/Partially Diagnosed/Not Diagnosed	US Result during Pregnancy	Cases (n)
I. fully diagnosed (n = 45)	corpus callosum dysgenesis/agenesis	45
II. partly diagnosed (n = 8)	ventriculomegaly	4
	microcephaly	2
	subarachnoideal cyst	2
III. not diagnosed (n = 11)	postnatal MRI	1
	postnatal cranial US	1
	autopsy	1
	neonatological finding	8

Abbreviation: ultrasound (US).

**Table 3:** Three years follow-up of the children affected with corpus callosum abnormalities (n = 20).

No	Genetic Result	Follow-Up	Motoric Complications	Seizure	Mental Complications
1	46,XY	affected	hemiparesis, right side		
2	46,XX (later: Aicardi syndrome)	affected		epilepsy	
3		affected			mental retardation, moderate
4		healthy			
5		affected			retardation
6		affected			slow development
7	47,XX+18	affected			mental retardation, mild
8		affected	asymmetric tone		
9		affected	hypotonic muscle, distal	epilepsy	
10		affected			slow development
11	47,XY+18	affected			slow development
12		affected			slow development
13		affected			mental retardation, severe
14		affected			slow development
15		affected		epilepsy	mental retardation
16		affected			slow development
17		affected			mental retardation, moderate
18		affected	tetraparesis	epilepsy	mental retardation, severe
19		affected			slow development
20	Wolf–Hirschhorn syndrome	affected	general hypotony		mental retardation, severe

## Data Availability

The datasets used and/or analyzed during the current study are available from the corresponding author upon reasonable request due to reason the data are not publicly available due to privacy and ethical restrictions.
